# Electrical Field-Assisted Gene Delivery from Polyelectrolyte Multilayers

**DOI:** 10.3390/polym12010133

**Published:** 2020-01-06

**Authors:** Yu-Che Cheng, Shu-Lin Guo, Kun-Da Chung, Wei-Wen Hu

**Affiliations:** 1 Proteomics Laboratory, Department of Medical Research, Cathay General Hospital, Taipei 10630, Taiwan; yccheng@cgh.org.tw; 2 Department of Biomedical Sciences and Engineering, National Central University, Zhongli District, Taoyuan City 32001, Taiwan; 3 School of Medicine, Fu Jen Catholic University, New Taipei City 24205, Taiwan; achillesguo@yahoo.com.tw; 4 Department of Anesthesiology, Cathay General Hospital, Taipei 10630, Taiwan; 5 Department of Anesthesiology, Tri-Service General Hospital and National Defense Medical Center, Taipei 11490, Taiwan; 6 Department of Chemical and Materials Engineering, National Central University, Zhongli District, Taoyuan City 32001, Taiwan

**Keywords:** layer-by-layer assembly, electrical field, polypyrrole, gene delivery, polyelectrolyte multilayer

## Abstract

To sustain gene delivery and elongate transgene expression, plasmid DNA and cationic nonviral vectors can be deposited through layer-by-layer (LbL) assembly to form polyelectrolyte multilayers (PEMs). Although these macromolecules can be released for transfection purposes, their entanglement only allows partial delivery. Therefore, how to efficiently deliver immobilized genes from PEMs remains a challenge. In this study, we attempt to facilitate their delivery through the pretreatment of the external electrical field. Multilayers of polyethylenimine (PEI) and DNA were deposited onto conductive polypyrrole (PPy), which were placed in an aqueous environment to examine their release after electric field pretreatment. Only the electric field perpendicular to the substrate with constant voltage efficiently promoted the release of PEI and DNA from PEMs, and the higher potential resulted in the more releases which were enhanced with treatment time. The roughness of PEMs also increased after electric field treatment because the electrical field not only caused electrophoresis of polyelectrolytes and but also allowed electrochemical reaction on the PPy electrode. Finally, the released DNA and PEI were used for transfection. Polyplexes were successfully formed after electric field treatment, and the transfection efficiency was also improved, suggesting that this electric field pretreatment effectively assists gene delivery from PEMs and should be beneficial to regenerative medicine application.

## 1. Introduction

Because oppositely charged polyelectrolytes can adsorb to each other through electrostatic interaction, they can form multilayers through layer-by-layer (LbL) assembly. This versatile technique can be utilized for drug delivery because the release of polyelectrolytes depends highly on the structure and composition of polyelectrolyte multilayers (PEMs) [1]. Drug delivery through PEMs give good spatial control; thus, this method can be applied to different medical devices such as stents [2,3], intraocular lens [4], dental implants [5], tissue engineering scaffolds [6,7], wound dressings [8,9], and bone grafts [10,11], by which not only drugs can be locally delivered to the target sites but their therapeutic effects can also be extended through the continuous releases.

Nucleotides, such as siRNA and DNA, are especially suitable for controlled delivery through PEMs because these anionic biomacromolecules can be immobilized with polycations easily through LbL assembly [12,13,14,15,16]. Polycations, such as chitosan, polylysine, and polyethylenimine (PEI), are frequently used as nonviral vectors due to their complexation and cell internalization capability. Consequently, PEMs composed of DNA and polycations have been used for substrate-mediated gene delivery [14,15,16,17]. Once DNA and polycations are released, they can spontaneously form polyplexes which can be internalized by cells for gene delivery purposes. Furthermore, the continuous erosion of PEMs in aqueous environments demonstrates sustained delivery [12]. In contrast to the transit transfection through direct polyplex administration, gene delivery through PEMs can elongate transgene expression [17]. Additionally, in situ transfection through PEMs delivery can be localized, suggesting that PEMs-mediated gene delivery gives excellent spatial and temporal control [18]. Although the drugs immobilized within PEMs can be slowly released, the entanglement between polyelectrolytes only allows partial drug delivery [19]. Our previous study also indicates that less than half of the loading gene can be released from PEMs [12]. Therefore, how to efficiently deliver the immobilized gene from PEMs is a challenge.

Because polyelectrolytes are bound in PEMs through electrostatic interaction, different stimuli, such as pH [20] and temperature [21], have been applied to induce PEM decomposition. An electrical field has also been used to promote polyelectrolyte release from PEMs. For example, Ayter et al. have applied hydrolytically degradable polycations to compose PEMs with DNA [22]. Because these polycations are sensitive to pH changes, electrolysis produces OH^−^ to trigger PEM disruption, which eventually releases DNA to aqueous environments. However, this switch-type manipulation exhibits quick DNA release, which is unsuitable to long-term delivery for regenerative medicine purposes [23]. On the other hand, our previous study also applied electrical fields to promote the deposition of DNA/chitosan multilayers, which suggested that electrical fields can lead the electrophoresis of polyelectrolytes, by which the loading efficiency can thus be improved [24]. These studies indicated that electrical fields can manipulate the stability of PEMs through electrochemical reaction and electrophoresis of polyelectrolytes, which should be a promising strategy to enhance the dissolution of polyelectrolytes from PEMs.

Therefore, we would like to improve the delivery efficiency of PEMs composed of DNA and PEI through the pretreatment of electrical fields in this study. Different electrical fields were investigated to determine an effective mode of operation. In addition, the effects of treating parameters were comprehensively evaluated for optimization. The surface morphology of treated PEMs was also examined to elucidate the mechanism of polyelectrolyte dissolution. Finally, the released supernatant under the electrical field was applied to in vitro experiments to determine whether the promoted gene delivery through this electrical field pretreatment can be applied for transfection purposes.

## 2. Materials and Methods

### 2.1. Materials

Branched PEI with molecular weights of 25 and 750 kDa as well as 2,4,6-trinitrobenzenesulfonic acid (TNBSA) were purchased from Sigma-Aldrich (St. Louis, MO, USA). Dulbecco’s modified Eagle medium (D-MEM), trypsin-EDTA, fetal bovine serum (FBS), phosphate-buffered saline (PBS) were obtained from Gibco (Carlsbad, CA, USA). Pyrrole and ammonium persulfate (APS) were purchased from Acros (Geel, Belgium) and Showa (Tokyo, Japan), respectively.

### 2.2. Plasmid DNA Preparation

Plasmid DNA encoding enhanced green fluorescent protein (pEGFP-C3) was purchased from Clontech (Mountain View, CA, USA). These plasmids were cloned and amplified using competent cells *Escherichia Coli* DH5α (Invitrogen, Carlsbad, CA, USA). The amplified plasmid DNA was isolated using sodium hydroxide and was extracted by chloroform and phenol. Restriction enzyme mapping, polymerase chain reaction (PCR) detection, and DNA sequencing were utilized to confirm the quality of purified plasmid DNA.

### 2.3. The Preparation of Polypyrrole (PPy) Films

**Figure 1:**
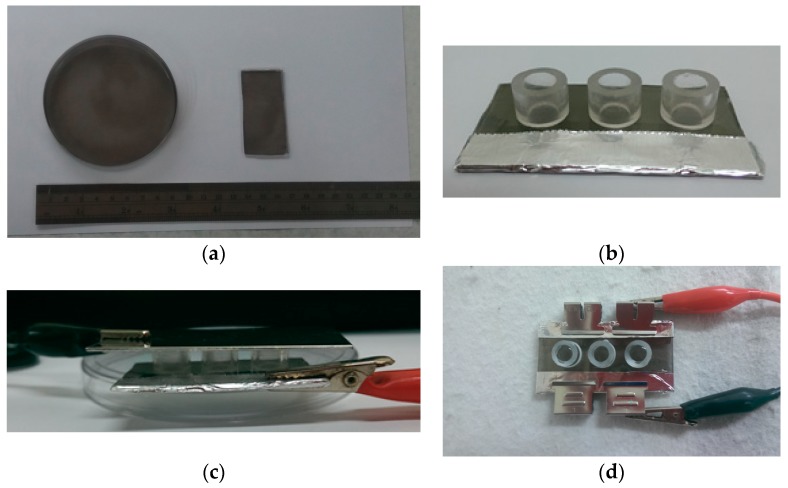
The electrical field administration to PEMs. (**a**) Polypyrrole (PPy) was deposited on polystyrene surfaces to form a conductive substrate, which was trimmed in the dimension of 6 by 3 cm. (**b**) Glass rings with an inner diameter of 6 mm and a height of 7 mm were glued on PPy films. (**c**) For the vertically electrical field, PBS was filled in the wells without bubbles, and the stainless steel counter electrodes were placed on the top of the wells. (**d**) For the horizontally electrical field, electrodes were placed at two opposing sides of the PPy films.

Polystyrene dishes with a diameter of 10 cm (Nunc, Penfield, NY, USA) were used as the substrate for PPy film deposition. Pyrrole and APS aqueous solutions were prepared in concentrations of 0.5 and 0.1 M, respectively. Equal volume (15 mL) of pyrrole and APS solutions were added to dishes with gently mixing at 4 °C. After 20 min incubation, these films were washed with deionized (DI) water and dried at 37 °C overnight. These PPy films were trimmed in the dimension of 6 by 3 cm and were cleaned by 70 wt% of ethanol aqueous solution before PEMs fabrication (Figure 1a).

### 2.4. Layer-by-Layer Assembly of PEMs

Glass rings with 6 mm in inner diameter and 7 mm in height were glued onto PPy film surfaces as wells for the preparation of the LBL assembly of PEMs (Figure 1b). In this study, PEI with a molecular weight of 25 kDa was applied for PEM preparation because of its good transfection ability [25]. However, these PEI molecules were incapable of being stably deposited due to their low molecular weight. Therefore, viscous 750 kDa PEI was firstly coated to form stable cationic surfaces on PPy films [14]. We placed 100 μL of 750 kDa PEI (2 g/L in PBS) per well. After a 2-h incubation, these PEI solutions were removed and rinsed by PBS thrice. Afterward, 4 bilayers of DNA/PEI were deposited by cyclically dipping 0.4 g/L of DNA and 25 kDa PEI solutions in volumes of 50 μL for 20 min, so that the theoretical amine to phosphate ratio (N/P ratio) was equal to 7.7 [26], and subsequently rinsed thrice with PBS between steps. 

### 2.5. The Pretreatment of Electrical Fields to Promote DNA and PEI Release from PEMs

Electrical fields were applied vertically and horizontally to PEMs to investigate their effects on destabilizing PEMs. For the vertically electrical field treatment, conductive PPy films were used as working electrodes. After filling PBS into the wells without bubbles, stainless steel was placed on the top of the wells as counter electrodes (Figure 1c). Regarding the horizontally electrical field treatment, electrodes were placed at two opposing ends of the conductive PPy films (Figure 1d).

After electrical field pretreatment, the dissolutions of DNA and PEI from PEMs in PBS were evaluated at different time points. Because amines of PEI can react with TNBSA to form chromogenic products, 20 μL of TNBSA (0.01 vol% in sodium bicarbonate buffer, pH 8.5) was added to 40 μL of samples [27]. After 1.5 h incubation at 37 °C, 20 μL of 10% SDS aqueous solution and 10 μL of 1 N HCl was added to terminate the reaction. The absorbance at 335 nm was determined by spectrometry (Synergy H1m, Biotek, Winooski, VT, USA), which were compared to the calibration curve determined by the results of standard PEI solutions to calculate the release of PEI. For DNA evaluation, samples were directly measured at the absorbance of 260 nm because PEI does not contribute any absorption at this wavelength [14].

### 2.6. Surface Morphologies of PEMs

Surface morphologies of PEMs were examined using scanning electron microscopy (SEM, S-3500N, Hitachi, Tokyo, Japan). Freeze drying was applied to dehydrate PEMs, and gold sputtering was applied before SEM examination. In addition, atomic force microscopy (AFM, SPA400 Seiko, Chiba, Japan) was also performed to quantify roughness. The images of wet PEMs were captured with a scan range of 10 × 10 μm in tapping mode.

### 2.7. Characteristics of Polyplexes Formed by PEMs-Released DNA and PEI 

After electrical field treatment, PBS was applied to release DNA and PEI in supernatant for 48 h. The released DNA and PEI from PEMs can form polyplexes due to their electrostatic interaction. To confirm whether the released DNA from PEMs was completely complexed by the released PEI, a gel retardation experiment was performed. The supernatant was collected and loaded in 1% agarose gels to perform electrophoresis at 100 V for 40 min, and the DNA location was illustrated using ethidium bromide staining [26]. Dynamic light scattering (DLS) analysis was performed to investigate the diameters and zeta potentials of the complexed nanoparticles (Zetasizer Nano ZS, Malvern, Worcestershire, UK).

### 2.8. Cell Transfection

To determine whether the released DNA/PEI nanocomplexes may be applied for transfection, an in vitro experiment was performed. After preparing PEMs in wells, DMEM with 10% of FBS was filled without bubbles for electrical field treatment. After a release for 48 h, the supernatants mixed with 12,500 of NIH/3T3 cells were seeded per well. The expression of pEGFP-C3 from transfected cells was examined using fluorescence microscopy (Eclipse Ti-U, Nikon, Tokyo, Japan). The intensities of fluorescence were quantified using an image software (NIS Elements Basic Research, Nikon, Tokyo, Japan).

### 2.9. Statistical Analysis

The statistical analyses were performed with SPSS (Chicago, IL, USA). A two-tailed Student’s *t*-test was performed to make comparison, and the errors were reported as standard deviations.

## 3. Results and Discussion

### 3.1. The Effects of Electrical Field on the Dissociation of LbL Multilayers

Because the assembly of PEM is based on the electrostatic interaction between polyelectroelectolytes, ions in aqueous solutions may facilitate the dissociation of PEM from substrates [28]. However, the passive dissociation is limited due to not only electrostatic interaction but also the entanglement between polymer chains [12,24]. Therefore, an external force may be beneficial to destabilize the PEM structure and promote the following release.

**Figure 2:**
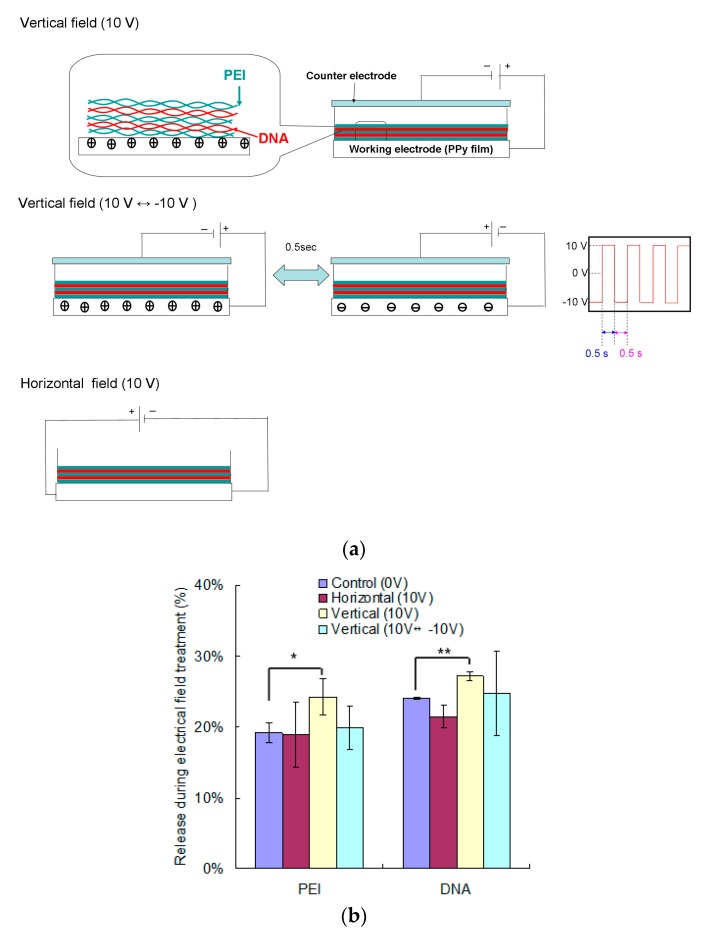

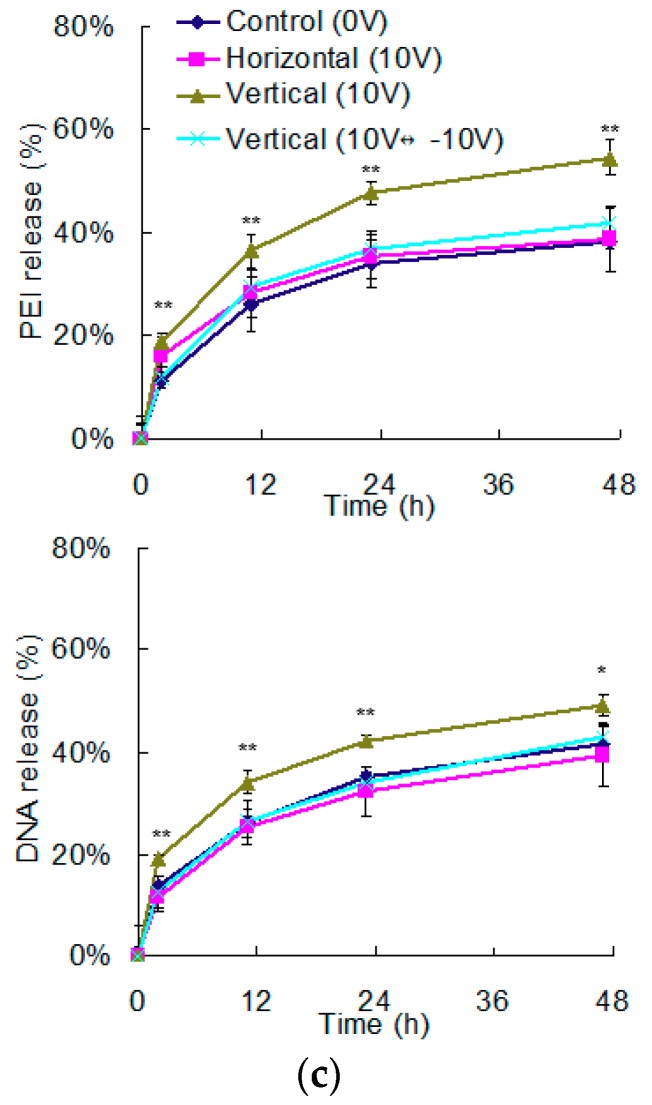
The effects of electrical field modes on polyelectrolyte delivery from PEMs. (**a**) Schemes of three different modes of electrical field. (**b**) The releases of PEI and DNA during 1 h treatment of different electrical fields. (**c**) PEMs with 1 h treatment of electrical fields were monitored for their following delivery in aqueous environments for 48 h (n = 3; *t*-test compared to the results of the control group at the same time, * *p* < 0.05; ** *p* < 0.01).

Electrical fields were investigated in this study to elucidate their effects on promoting the dissociation of PEMs. Conductive PPy films were applied as substrates, and PEI/DNA was deposited as four double layers, i.e., (PEI/DNA)_4_, which led the total loads of PEI and DNA 5.0 and 5.9 μg per well, respectively. Three different molds of electrical fields were firstly investigated (Figure 2a). For vertically electrical fields, direct current (DC) power supply was either administrated positive bias continuously (10 V) or biphasically (10 V ↔ −10 V as square waves of 1 Hz with a duty cycle of 50%). Regarding the horizontally electrical field, constant 10 V was applied through electrodes at two opposing sides of the PPy films. 

The electrical field treatments were applied to PEMs for 1 h, and the released PEI and DNA were determined spectrometrically (Figure 2b). The results showed that only the vertically electrical field (10 V) induced significant release of DNA and PEI from PEMs during the treatment. Then, we kept these PEMs in PBS to continuously evaluate their following release (Figure 2c). For the control group (0 V), although the release rates of both DNA and PEI decreased over time, the releases continued for 48 h. Regarding the electrical field treated groups, their delivery patterns were similar to that of the control group. Compared to the control group, horizontal electrical field and biphasically vertically electrical fields (10 V ↔ −10 V) did not promote delivery, no matter of PEI or DNA. In contrast, the constant vertically electrical field (10 V) significantly improved both PEI and DNA releases from PEMs. In addition, the release difference, especially the PEI results, increased over time. These results suggested that 1 h treatment of the vertically electrical fields in constant voltage seemed to loosen the LBL structure, which therefore not only induced PEM dissolution in the initial stage but also extended the releasing effect for long-term delivery. Therefore, we would like to use the electrical field as a pretreatment in the following experiment to avoid the burst release of polyelectrolytes (Figure 2b).

The different behaviors of polyelectrolyte delivery may correlate to not only electrophoresis but also electrochemical effects. Regard the constant vertical field, the PPy film, i.e., the substrate of the PEMs, was applied as an anode. Its positive charges on surfaces may push PEI outward, and our results suggested that the releases of DNA and PEI were both improved. Actually, we tried to reverse the bias by PPy films as the cathode, but the release of polyelectrolytes was not enhanced (data not shown). Considering the molecular weights of polyelectrolytes, plasmid DNA was in range of mega Dalton whereas PEI was only 25 kDa. It should be easier to move small PEI molecules by electrical fields than the large DNA molecules. Further, the branched PEI was in a compact morphology, so its low entanglement made it easy to move in PEMs [29]. In addition, DNA and polycations in PEMs may assemble as polyplexes within PEMs [18,30], and thus the vertical field was capable of moving these cationic nanocomplexes out from PEMs.

Regarding electrochemical concerns, the oxidation of water molecules may protonate DNA molecules to reduce their negative charges [31]. In addition, chloride ions in PBS can be oxidized at anodes to produce chlorine, which may react with water to form HClO. Because amines of PEI can react with HClO to form monochloramines, this reaction also decreased the positive charges of PEI [32]. These two electrochemical reactions reduced the charge density of polyelectrolytes, so the electrostatic interaction was interfered to eventually destabilize the PEM structures. To ensure our hypothesis, the biphasically electrical field was also performed, and this treatment did not promote polyelectrolyte release because the electrochemical reaction cannot stably occur under this quickly switching electrical field. It suggests that electrochemical reactions are critical to regulate the stability of PEMs. Regarding the horizontal field, we hypothesized that substrate-mediated current may drive the movement of polyelectrolytes in PEMs toward opposite ends. However, this horizontal movement was likely in vain in polyelectrolyte delivery. In addition, electrochemical reaction did not take place because the horizontal field only led current pass through the conductive substrate. Overall, these results suggest that the vertically electrical fields in constant voltage can be applied to destabilize PEMs to promote the release of polyelectrolytes.

### 3.2. The Regulation of Polyelectrolyte Delivery through the Parameters of Vertically Electrical Fields

**Figure 3:**
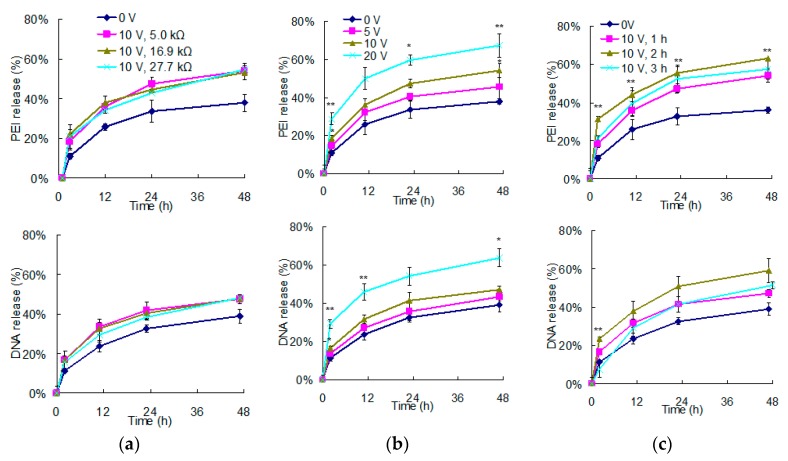
The effects of the parameters of the vertically electrical field pretreatment on polyelectrolyte delivery from PEMs. (**a**) Ten voltage of electrical fields pretreated PEMs on PPy films with different electrical resistances for 1 h to determine the effect of electric currents. (**b**) Different voltages pretreated PEMs for 1 h to determine the effect of intensity of electrical fields. (**c**) Electrical field in 10 V pretreated PEMs for different durations to determine the effect of treatment duration. These electrical field-treated PEMs were monitored for their following delivery in aqueous environments for 48 h. (*n* = 3. For (**b**), *t*-test compared to the results of the 5 V pretreating group at the same time, * *p* < 0.05; ** *p* < 0.01. For (**c**), *t*-test compared to the results of the 1 h pretreated group at the same time, ** *p* < 0.01.).

Considering the promotion effects of vertically electrical fields on polyelectrolyte delivery from PEMs, we would like to further investigate its control parameters. In this study, PPy films were applied as electrodes because they are not only chemically stable but also have electrical resistances controllability [33]. Pyrrole in concentrations of 0.3, 0.4, and 0.5 M were polymerized to fabricate PPy films with electrical resistances of 27.7, 16.9, and 5.0 kΩ, respectively. Because a constant 10 V of the vertically electrical field was administrated, the higher electrical resistances of PPy films resulted in lower electric currents. Therefore, 1 h of a vertically electrical field was applied to PEMs to investigate the following releases (Figure 3a). Compared to the control group, both DNA and PEI were enhanced of their releases under vertically electrical fields. However, there was no difference among these experimental groups, suggesting that the release of polyelectrolytes from PEMs should be independent of the electric currents.

In contrast, when we used the same PPy films (5.0 kΩ) and only adjusted the voltages of the vertical fields to pretreat PEMs, both DNA and PEI releases from PEMs increased with voltages, suggesting that the intensity of the vertical field highly impacted the stability of PEMs (Figure 3b). Similar results have also been reported by Aytar et al. [22]. They applied poly (β-amino easter)s and plasmid DNA to deposit PEMs on electrodes using LbL assembly. Negative bias was applied to electrolyze water for OH- production, by which degradation of poly (β-amino easter)s were promoted. Their results showed that the higher the voltages, the faster the PEMs hydrolyzed. Therefore, electrochemical reaction rates can be easily manipulated through the levels of electrical fields, by which polyelectrolytes delivered from PEMs are thus controlled according to requirements.

In addition to the control parameters of electrical fields, we were also curious about the effect of treatment duration on polyelectrolyte delivery. Vertical fields were applied to PEMs for 1, 2, or 3 h, and the following releases of polyelectrolytes were continuously monitored for 48 h (Figure 3c). Compared to the control group which demonstrated only 36% of PEI release and 39% of DNA release, 1 h of vertical field administration increased the releases of PEI and DNA to 54% and 47%, respectively. The 2 h administration further increased the releases of PEI and DNA to 63% and 59%, respectively. These results suggest that the elongation of electrical field pretreatment should sustain electrochemical reactions to facilitate the level of destabilization. However, when the vertical field was elongated to 3 h, the release of PEI and DNA were only 58% and 51%, respectively. Bubbles, probably oxygen or chlorine [31,32], were found around the electrodes, suggesting that too long a treatment time may cause gas insulation.

To avoid 20% to 30% burst releases of DNA and PEI from PEMs in the first hour (Figure 2b), an electrical field was applied as a pretreatment and cells may be transfected by the DNA and PEI released afterward. According to the abovementioned experiments, increasing voltages and treatment time both effectively destabilized PEMs to eventually increase DNA and PEI delivery. Among them, the 20 V for 1 h and 10 V for 2 h groups demonstrated the best promotion, which showed the PEI releases of 69% and 63%, and the DNA releases of 64% and 59%, respectively. Considering that only 70% to 80% of DNA and PEI remained on PEMs after discarding the first hour-release, our results indicated that the destabilization through electrical field pretreatment promoted the release of most electrolytes from PEMs.

### 3.3. Surface Morphology of Electrical Field Treated PEMs 

**Figure 4:**
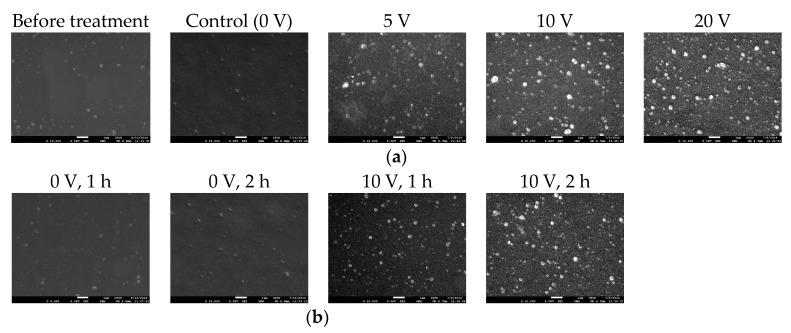
Scanning electron microscopy analysis of PEMs. (**a**) Surfaces of PEMs before or after treating different voltages for 1 h. (**b**) Surfaces of PEMs in aqueous environments with or without electrical field treatment for 1 or 2 h. (scale bar = 1 µm).

**Figure 5:**
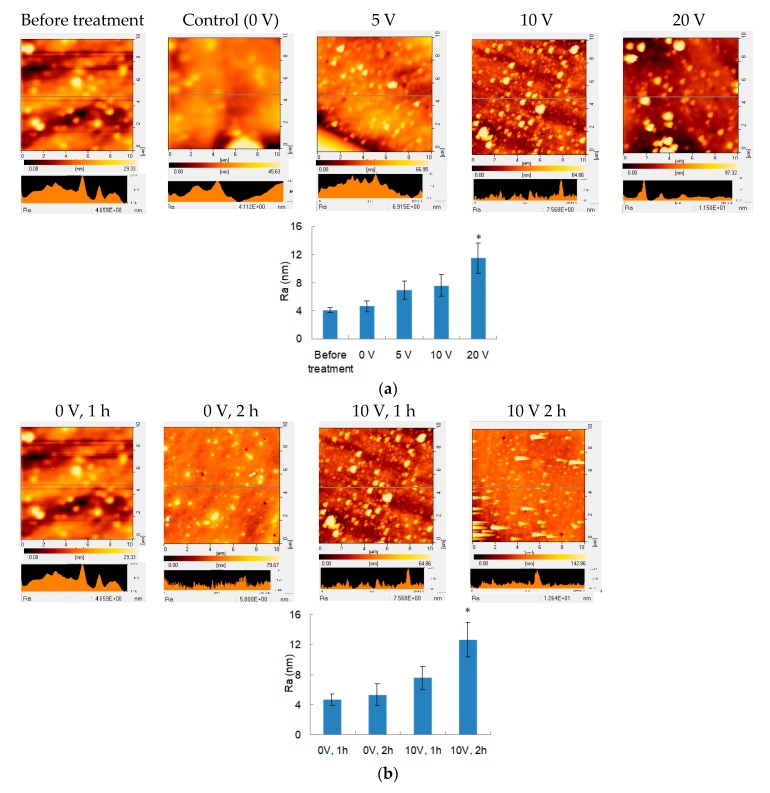

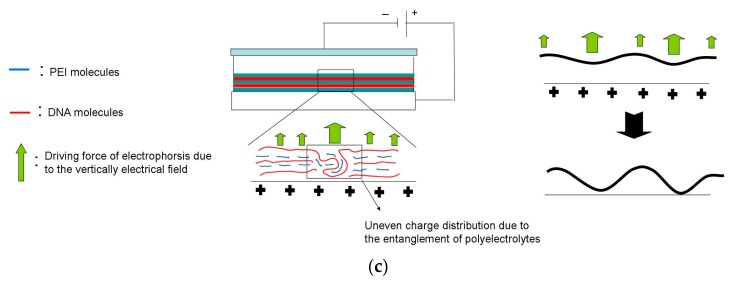
Atomic force microscopy analysis of PEMs. (**a**) Surfaces of PEMs before or after treating different voltages for 1 h. (**b**) Surfaces of PEMs in aqueous environments with or without electrical field treatment for 1 or 2 h. Roughness averages (Ra) were also determined (*n* = 3, and *t*-test compared to the 0 V group, * *p* < 0.05) (**c**) The explanation scheme of surface roughness after vertically electrical field treatment. Entanglement of polyelectolytes caused uneven charge distribution, and these unstable regions were susceptible to electrical fields to demonstrated quick release. In addition, the thinner regions became closer to the electrode, which thus were released faster to eventually form bumps and nanoparticles on the PEM surfaces.

Since the electrical field treatment is speculated to induce electrophoresis and electrochemical reaction, PEMs may be destabilized. Therefore, SEM analysis was performed to evaluate the surface morphology of PEMs immediately after electrical field treatment (Figure 4). Before incubation in PBS, the PEMs exhibited a smooth surface. For the PEMs immersed in PBS for 1 h without treating electrical field, the surface was also relative smooth. When different electrical fields were applied to PEMs, bumps and nanoparticles seemed to increase with voltages (Figure 4a). Therefore, the tapping mode of AFM was further applied to examine these PEMs, and the higher the treating voltage, the rougher the treated surface (Figure 5a). Treatment time also demonstrated similar trends: as the treatment time was elongated to 2 h, the surface roughness was significantly increased compared to that treated for 1 h (Figure 4b and Figure 5b).

The surface morphology results indicated that PEMs increased roughness after the treatment of electrical field. The roughness represents the height difference. Although PEMs were prepared through LbL assembly, even distribution of these charged macromolecules is difficult as entanglement may occur during deposition, which frequently leads uneven charge distribution to form unstable structures (Figure 5c). Therefore, these susceptible regions were superior in polyelectrolyte release under electrical fields to preferentially decrease the thickness of PEMs. When these polyelectrolyte-dissolved regions became thinner and close to the electrodes, the effects of electrophoresis and electrochemical reactions were highly promoted in these thin regions to further increase the roughness of surface morphology.

### 3.4. The Release of Polyelectrolyte from PEMs for Transfection Application

**Figure 6:**
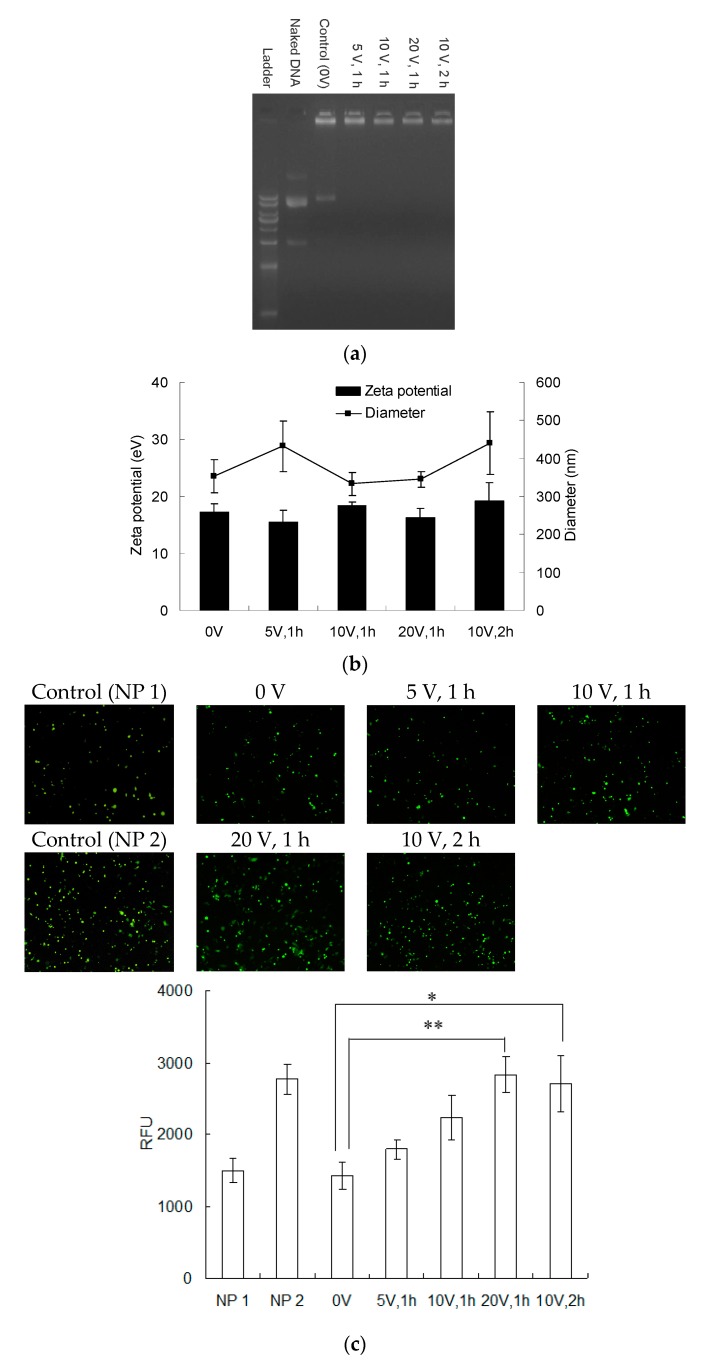
The release of DNA and PEI from PEMs for transfection application. After electrical field pretreatment, PEMs were kept in PBS for 48 h and the supernatant was collected. (**a**) The supernatant was loaded to agarose gel for electrophoresis to determine the complexation efficiency of PEI to DNA. (**b**) To determine the sizes and surface charges of DNA/PEI polyplexes in supernatant, DLS analysis was performed. (**c**) The released polyplexes were applied to transfect NIH/3T3 cells. Because eGFP was encoded in plasmid DNA, transfected cells expressed green fluorescence. In addition, directly mixed DNA and PEI for complexation were applied as the control groups NP 1 and NP 2 to confirm whether the transfection efficiencies of DNA and PEI delivered from PEMs were similar to those prepared by the traditional complexation method. According to the results in Figure 4b, the NP 1 control group was prepared using DNA of 2.34 μg and PEI of 1.89 μg and the NP 2 control group was prepared using DNA of 3.82 μg and PEI of 3.46 μg, which were equal to those released from PEM pretreated by 0 V and 20 V, respectively. Fluorescent microscopy was performed after transfecting for 3 days, and the fluorescent images were analyzed using image software (scale bar = 200 μm; *n* = 3, *t*-test compared to the results of 0 V group, * *p* < 0.05; ** *p* < 0.01).

Since electrical field pretreatment promoted DNA and PEI delivery from PEMs, we further investigated whether these released DNA and PEI were capable of applying to transfection. Therefore, PEMs were kept in PBS to continuously release for 48 h after the electrical field treatment, and the supernatant was collected for analysis. A gel retardation analysis was applied to determine whether the released DNA and PEI were completely complexed to each other (Figure 6a). The results showed that DNA released from PEMs without electrical pretreatment (0 V) was incompletely complexed. In contrast, the electrical field pretreated groups all demonstrate perfectly complexation. The release promotion of electrical fields was more obvious on PEI than DNA (Figure 4b). The control group without electrical field pretreatment (0 V) released 2.34 μg of DNA and 1.89 μg of PEI, which only resulted in the N/P ratio of 6.1. In contrast, the PEMs pretreated 20 V for 1 h group eventually led 3.82 μg of DNA and 3.46 μg of PEI delivery, which gives the N/P ratio of 7.0. Therefore, the electrical field pretreatment not only increased DNA and PEI release but also raised the N/P ratio to facilitate complexation efficiency.

The size and surface charges of nanoparticles in the supernatant were investigated using DLS analysis (Figure 6b). The results showed that polyplexes were formed in sizes between 300 to 500 nm with zeta potentials between 15 to 20 eV, and there was no difference between the control (0 V) and experimental groups, suggesting that the electrical field-assisted PEI and DNA releases formed similar nanocomplexes as those from passive delivery (0 V).

Afterward, these delivered PEI and DNA were applied to treat NIH/3T3 cells to evaluate their transfection efficiencies (Figure 6c). In this study, directly mixed DNA and PEI for complexation were applied as the control groups NP 1 and NP 2 to confirm whether the transfection efficiencies of DNA and PEI from PEMs were similar to those prepared by the traditional complexation method. According to the results in Figure 4b, the NP 1 group was prepared using DNA of 2.34 μg and PEI of 1.89 μg DNA and the NP 2 group was prepared using DNA of 3.82 μg and PEI of 3.46 μg, which were equal to those released from PEM pretreated by 0 V and 20 V, respectively. Because plasmid DNA encoding eGFP was applied in this study, transfected cells exhibited green fluorescence. The image results showed that the higher voltage and the longer treating times all resulted in the higher transfection efficiency, which was consistent with the delivery trend. In addition, the transfection results of NP 1 and NP 2 were similar to those of the 0 V and 20 V groups, respectively. These results indicated that the DNA and PEI released from electrical field-pretreated PEMs owned comparable transfection capacity as those prepared by the traditional complexation method.

In our previous study, we applied LbL assembly to prepare PEMs of DNA and nonviral vectors [12,14,17]. Our results showed that PEMs may sustain the deliveries of these polyelectrolytes. Because genes can be continuously delivered from PEMs to transfect cells, the expression of transgene was thus elongated. To increase the delivery efficiency, an electrical field was applied as a pretreatment in this study, and our results indicated that DNA and PEI delivery from PEMs were both promoted after electrical field treatment, suggesting its potential on gene delivery application.

## 4. Conclusions

In this study, an electrical field was investigated as a pretreatment to assist the delivery of polyelectrolytes through PEMs. Electrical fields in a vertical direction significantly improved the delivery of both polycations and polyanions. The promotion effects were adjustable in that a higher voltage and a longer treatment time resulted in greater delivery. In addition, the increasing roughness of the PEMs after electrical field treatment also suggested the destabilization effects of electrical fields on PEMs structures. This enhancement can be used for gene delivery to improve transfection efficiency and elongate transgene expression, which should be beneficial to tissue engineering applications.
